# The effectiveness of Realistic Mathematics Education approach: The role of mathematical representation as mediator between mathematical belief and problem solving

**DOI:** 10.1371/journal.pone.0204847

**Published:** 2018-09-27

**Authors:** Putri Yuanita, Hutkemri Zulnaidi, Effandi Zakaria

**Affiliations:** 1 Department of Mathematics Education, Faculty of Teacher Training and Education, University of Riau, Pekanbaru, Indonesia; 2 Department of Mathematics and Science Education, Faculty of Education, University of Malaya, Kuala Lumpur, Malaysia; 3 Department of Innovation in Teaching and Learning, Faculty of Education, National University of Malaysia, Bangi, Selangor, Malaysia; University of California Irvine, UNITED STATES

## Abstract

This study aims to identify the role of mathematical representation as a mediator between mathematical belief and problem solving. A quasi-experimental design was developed that included 426 Form 1 secondary school students. Respondents comprised 209 and 217 students in the treatment and control groups, respectively. SPSS 23.0, ANATES 4 and Amos 18 were used for data analysis. Findings indicated that mathematical representation plays a significant role as mediator between mathematical belief and arithmetic problem solving. The Realistic Mathematics Education (RME) approach successfully increased the arithmetic problem-solving ability of students.

## Introduction

Education equips younger generations with important skills and knowledge. Effective learning enables students to learn through creative teaching methods and acquire knowledge in class; the latter becomes an exciting activity through the effort of teachers [1]. Mathematics education motivates students to become critical and innovative and to cultivate sound reasoning in problem solving. Mathematics education is an active, dynamic and continuous process; activities in mathematics education help students develop their reasoning, think logically, systematically, critically and thoroughly and adopt an objective and open attitude when dealing with problems [2]. Teaching and learning consist of three main components, namely, teachers, students and content. Students must be equipped with knowledge and high-level skills and teachers must possess knowledge and professionalism. Problem-solving skills enable students to think creatively and critically by using progressive and challenging thought processes; creative and critical thinking will help develop a nation and address its needs [3]. Teaching and learning processes in the classroom serve as a study ground for researchers. A future educator can determine effective teaching methods through this process. Teachers and students in Indonesia acknowledge the need to improve the current status of teaching and learning mathematics. Since 1970, Indonesia has applied a modern approach towards teaching mathematics. However, this approach has created problematic situations in various schools.

Mathematics learning in Indonesia remains below average compared with developing countries in Asia, such as China, Singapore and Malaysia [4]. In the past, China surpassed other western countries in internationally scaled mathematics achievement, such as in PISA and International Mathematical Olympiads (IMO) [5]. One of the challenges faced by mathematics teaching is the constantly changing curriculum. Traditional mathematics teaching persists in secondary schools. If the paradigm is to be changed, then teachers must find a teaching and learning approach that is consistent with the constructivist approach. One of these teaching and learning approaches is Realistic Mathematics Education (RME), which was introduced in 2001 in Indonesia by the Realistic Mathematical Education of Indonesia (known as Pendidikan Matematik Realistik Indonesia or PMRI). The goal of PMRI is to revolutionise and improve mathematics education [6].

The RME approach was first developed by the Freudenthal Institute in the Netherlands in 1971. The RME approach for mathematics is widely known as the best and most detailed approach, which was expanded from the problem-based approach for mathematics education [7]. Teaching and learning RME have five main criteria, namely, students’ experience in daily life; changing reality to a model and changing the model through a mathematical vertical process before turning it into a formal system; use of students’ active style; use of discussions and question and answer methods to cultivate the mathematics skills of students and formation of a connection between concepts and topics until learning becomes holistic and complete [8]. Since 2001, many teachers in Indonesia have been trained to use the RME approach. RME has been implemented in 13 of 33 provinces. On the basis of this finding, a study is conducted to develop a teaching module that uses RME and to examine the effects of teaching and learning using the mathematics learning module for secondary schools in Indonesia. Teaching and learning via RME aim to solve the problems faced by teachers and students.

The purpose of RME is to transform mathematics learning into a fun and meaningful experience for students by introducing problems within contexts. RME starts with choosing problems relevant to student experiences and knowledge [4]. The teacher then acts as a facilitator to help students solve contextual issues. This contextual problem-solving activity brings positive impact to the mathematical representation of students, which is related to their problem solving skills [9,10]. The best way to teach mathematics is to provide students with meaningful experiences by solving the issues they face every day or by dealing with contextual problems. Realistic mathematics education enables the alteration of the mathematical material concept and its relationship. Realistic mathematics education changes the culture towards a dynamic one, but still in the corridor of the educational process. Therefore, realistic mathematics education is an innovative learning approach that emphasises mathematics as a human activity that must be associated with real life using real world context as the starting point of learning [11].

Mathematical belief is the key idea in the application of mathematical teaching approaches [12]. The mathematical belief of a student is formed from his or her attitude towards his or her mathematical knowledge, thereby enhancing one’s mathematical value. This view is supported by Anderson, Roger and Klinger [13], who found that positive mathematical belief influences the performance of secondary school students in Canada. According to The National College of Teachers of Mathematics (NCTM) [14], this belief influences the ability of students to evaluate their skills, desire to perform mathematical tasks and mathematical disposition. Knowledge of these steps is not enough in performing mathematical tasks because students must also believe in the truth of concepts and procedures. The mathematical belief of students consists of three main factors, namely, students’ belief in their ability, in the mathematical discipline and towards mathematical teaching and learning.

Hwang, Chen, Dung and Yang [15] defined representation as the process of turning a concrete model in the real world into an abstract concept or symbol. In mathematical psychology, representation refers to the relationship between objects and symbols. The five outer levels used by representation in mathematics education are real-world objects, multiple representation, arithmetic symbol representation, oral representation and picture or graphic representation. The last three representations are abstract and are considered high-level representation in solving arithmetic problems. Ratio with the aid of arithmetic symbol representation involves translating mathematical problems into arithmetic formulas. Language ability representation involves interpreting characters and relationships in mathematical problems into verbal or vocal forms. Picture or graphic representation involves interpreting mathematical problems into pictures or graphics. In this study, the mathematical representations applied by students consist of picture representations, graphic representations, tabular representations, symbolic representations, mathematical notes, written text representations, words and language.

Problem solving is one of the higher-order thinking skills that require students to think critically and creatively [16]. Ibrahim [17] claimed that the ability to solve problems involves the use of learned principles to solve problems to achieve certain meanings. In the present study, problem solving skills refer to the ability to solve problems given in the learning context using the RME approach. The problems are based on daily routines and real situations that students were previously aware of. Problem solving skills in this study refer to the ability of students to solve related concepts and procedures in arithmetic problems.

## Problem statement

Varying teaching styles increases the difficulty of learning and understanding mathematics. Moreover, students are afraid of mathematics [6]. The research object in mathematics is abstract and traditional teaching approaches are ill suited for such matters. The unsatisfactory understanding of mathematics and performances of students are attributed to several factors. Firstly, teachers dominate the learning process of a classroom by applying unidirectional and traditional teaching methods. According to Roberg [18], traditional learning focuses on skill and concept acquisition. Thus, this approach is unsuitable for improving problem solving skills. Secondly, teachers merely present theories and definitions. For example, a theorem is explained through examples and students are assessed through a series of exercises and questions. Teaching is the process of obtaining facts from definitions, attributes and formulas in the mathematics textbook of students. Teachers simply follow the steps given in textbooks without considering whether the process is correct or not. Thus, the learning process becomes mechanical, wherein teachers simply set formulas and solutions for students [19]. Findings on the application of modern mathematics show that mathematical learning is a low-value learning process [6].

Mathematical literacy in Indonesia cannot improve with the way mathematics is taught in schools. The current teaching approach does not focus on logical, analytical, systematic, critical and creative thinking among students; rather, teachers simply depend on textbooks [20]. This approach requires students to memorise the correct steps for answering questions. However, students encounter difficulty when they are given questions that cannot be solved using such steps. The students learn passively and memorise formulas without understanding what the questions actually mean. Thus, they do not benefit from what they are learning and often make mistakes. Zainal [21] stated that students prefer to memorise the formulas and steps provided by their teachers without comprehending the actual concept. Thus, students only know how to calculate, but they cannot solve everyday problems that involve a mathematical concept or skill. Many students perceive that mathematics is difficult to learn and requires a long time to gain understanding. Students are considered to have learned successfully when they can remember and restate facts or use them to answer questions in examinations. Thus, students have low understanding and mastery of mathematical concepts.

According to Taat, Abdullah and Talip [22], teachers must use an approach that deeply influences the understanding of students. Sabandar [23] pointed out the need for challenging settings and problems to encourage students to learn more than they used to. Mathematics is mainly problem solving-oriented. Thus, teachers have to connect mathematics with everyday problems. To improve the problem-solving skills of students, mathematics teachers must provide open, realistic problems with multiple probable answers [24]. In realistic mathematical learning that uses open problems, students use their problem solving methods and understand the methods used by others. This ability is important because mathematics is used in almost every aspect of life.

Few studies show the relationship between mathematical representations and solving mathematical problems. Hwang, Chen, Dung and Yang [15] mentioned that good problem-solving skills are the key to obtaining the exact solution to a problem. Gagatsis and Elia [25] studied the role of four-way representations, namely, verbal, decorative picture, informal picture and counting line representations, in solving mathematical problems. Students generally achieve better problem-solving skills when the four representation models are used than when the single-representation learning model is applied. Ling and Ghazali [26] found that symbols of numeric and arithmetic representations are the most frequently used models by students in solving problems; these symbols include answer verification from a whole set of questions. This study must be expanded to measure samples until the findings can be generalised. Representation assessment and problem solving strategies are needed to create a specific rubric. Hwang, Chen, Dung and Yang [15] studied the influence of the ability and creativity of various representations in mathematical problem solving using a multimedia whiteboard system. They found that the representation ability of various students is key to effectively solving mathematical problems. The study should be expanded from the aspect of research subjects until the findings can be generalised because the focus was not on the direct influence of representation and creativity on real-life problem-solving skills.

Mathematical belief is one of the components of the affective domain, which plays a critical role in mathematical learning. The affective aspect determines student success in learning mathematics and includes attitude, interest, self-concept and belief [27]. The NCTM revealed the roles of cognitive and affective aspects in mathematical learning [28]. Both aspects are influential in the mathematical performance of students. Student belief in mathematics can influence the view towards mathematical discipline, which is related to mathematical teaching and learning [3]. According to Kloosterman [29], many students have strong mathematical belief. Mathematical belief attracted the attention of many educational mathematics researchers, particularly in other countries. However, only a few studies were conducted in Indonesia on the mathematical belief of students. The mathematical belief of students can be improved through the teaching method applied by teachers. Lee, Zeleke and Mavrotheris [30] studied the development of student belief, which can be expanded to the influence of the students’ condition and setting. Greer, Verschaffel and de Corte [31] believed that the mathematical belief of students is influenced by teachers, textbooks, learning strategy and the use of problems that exist in their surroundings during learning activities. Interrelated factors influence changes in students’ mathematical belief. Therefore, all related factors should be considered to increase the mathematical belief of students.

Arithmetic is one of the mathematical learning topics applied in daily life. Students experience difficulty in understanding arithmetic-related problems. The concept acquired by students is not formed by the students solely. Hence, students fail to retain the concept in their memory. Once students learn a new concept, they forget the old one. Many students do not solve problems by understanding the concept and rely instead on intuition or memorisation. Many everyday problems can be solved using comparison to facilitate the selection of contextual problems as a first step of the learning process. This step enables students to form their concepts, principles and mathematical procedures related to the topic. In accordance with the objective of mathematical learning, which is to prepare students to use mathematics and its way of thinking in daily life, we attempt to develop an arithmetic module that fits the RME approach. According to Sunismi [32], the learning approach and increased cognitive development showed the presence of interaction in the understanding of mathematical concepts in solving problems for Form 2 secondary school students. Haji [33] mentioned the lack of significant interaction between the approach and ability of students to solve problems.

Other studies revealed the RME function in mathematics learning. The study unveiled the relationship among mathematical representation and belief and problem-solving skills. Warsito, Darhim and Herman [9] examined the effect of RME on improving mathematical representation ability. Meika, Suryadi and Darhim [10] applied RME in students’ errors in solving combinatoric problems. Yuanita and Zakaria [34] investigated the differences in the mathematical belief of students based on their abilities in RME and students enrolled in regular classes. The results of the previous study showed that RME can be effectively used to predict the mathematical representation, belief and problem-solving skills of students. A previous study suggested a highly effective learning approach in RME; this approach includes designing instructional materials in accordance with real-life contexts that train student thinking skills. Mathematical learning should be delivered in a form that gives students an opportunity to reinvent ideas and mathematical concepts along with teacher guidance through exploration of various contextual issues and the effects of RME on students’ attitude, problem-solving ability, learning interest or other variables related to mathematics learning.

Radzali, Meerah and Zakaria [35] examined the relationship between mathematical belief and representation with mathematical problem solving. Results show that mathematical belief and representation contributed to the problem solving of students. The findings of this study are important because no other study has examined the factors mentioned. A previous study focused on examining each separately stated factor. However, studies that incorporate all three factors into inside or outside of the country are lacking. Therefore, the current study attempts to investigate these three factors simultaneously to identify the effect of mathematical representation as a mediator between mathematical belief and problem solving.

The significance of this study is its emphasis on mathematical representation, mathematical belief and problem-solving skills, which are vital to building mathematical discipline. Mathematical representation and belief and problem-solving skills are often misconceived. Therefore, the use of RME in the classroom can provide examples for students based on their daily activities. This approach could assist them in mathematical representation and belief and improve their problem-solving skills. Thus, this study investigates the difference in mathematical representation and belief and problem-solving skills of students who learned with RME and students who were engaged in conventional learning. This study also investigated the effect of mathematical representation as a mediator between mathematical belief and problem solving.

**Fig 1:**
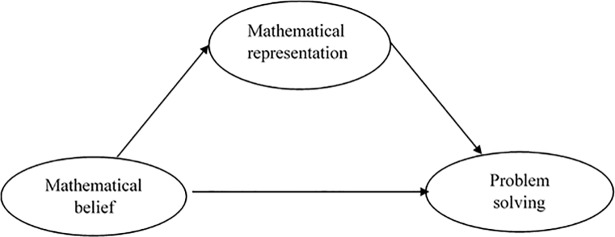
Conceptual framework of the study.

Fig 1 shows that this study was performed to identify the effectiveness of the RME approach in mathematical belief and representation and problem solving. In addition, this study identified the role of mathematical representation as a mediator between mathematical belief and problem solving. This study was conducted to answer the following research questions:

1. Does the use of the RME approach have any significant effect on mathematical belief, mathematical representation and problem solving?
2. Is mathematical representation a significant mediator between mathematical belief and problem solving?

## Methodology

### Participants

The study involved 426 Form 1 secondary school students, who were divided into control and treatment groups. RME and traditional approaches were used by 209 and 217 students, respectively. The treatment group had 95 male and 114 female students. Fifty-six students had low ability, 96 had average ability and 57 students had high ability. The control group had 103 male and 114 female students. Sixty of them had low ability, 96 had average ability and 61 students had high ability. The mathematics ability of students was based on the results of their mathematics achievement in the past semester. The results were then categorised using Anates software into low, moderate and high [36]. The demographic profile is shown in Table 1.

**Table 1:** Demographic profile.

Demographic		Treatment	Control
Gender	Male	95	103
	Female	114	114
Ability	Low	56	60
	Average	96	96
	High	57	61

### Research design

The study used the quasi-experimental design with non-equivalent pre- and post-test control groups. The control group was created for comparison with the experimental group [37,38]. The quasi-experimental design refers to an experiment that consisted of units with treatment. This approach was utilised because the study used the existing class [39], which indicated that the research subjects were not selected randomly [40]. The quasi-experimental design was used to determine the effectiveness of the RME approach in improving problem solving skills, mathematical representation and belief of students. The research design is shown in Table 2.

**Table 2:** Quasi-experimental research design.

Group	Pre-test	Treatment	Post-test
Treatment	O_1_	X_1_	O_2_
Control	O_3_	X_2_	O_4_

Details

O_1_, O_3_ = Pre-test for treatment and control groups

O_1,_ O_2_ = Post-test for treatment and control groups

X_1_ = Treatment with RME approach

X_1_ *=* Treatment with traditional approach

Pre- and post-tests were conducted in both groups. The pre-test ensured similarity between groups and statistical control by comparing the mean of mathematical belief, representation, and problem solving with significant value of more than 0.05. The treatment group was given a task using the RME approach in teaching, whereas the traditional method was used as control group. Students in both groups were taught during 10 two-hour sessions in their respective classrooms. The post-test was given to both groups after they were taught social arithmetic to determine the effectiveness of the RME approach. The test questions for pre- and post-tests were similar. The researcher observed each session for both groups throughout the discussion. Observations were conducted for 5 weeks in 10 sessions for both groups. A post-test was given to the two groups after social arithmetic and ratio were taught.

Internal and external validities were determined with reference to Johnso and Christensen [40]. Internal validity is a controlled variable set by the researcher that aims to identify the actual effect on the treatment variable. External validity sees how far the findings can be applied to individuals and settings other than the ones in the study. Issues, such as selection of research and lost subjects (mortality), emotional maturity, intellectual and physical well-being, testing, research instrument and validity of research objects, can arise from the quasi-experimental design of pre- and post-tests. These issues refer to factors related to the study and the attitude and emotion of students.

### Experimental group

The experimental group was taught using the RME approach. Teachers followed three main phases to teach this approach. In the first phase, teachers introduced realistic problems to students and helped them understand the problem setting. Teachers revised previous concepts and connected them with the experience of students. In the second phase, students worked in groups. Each student had a book that contained contextual questions and constructed situational problems, shared ideas, analysed patterns, made guesses and expanded problem-solving strategies based on knowledge or formal experience. The third phase of assessment showed the progress of students in problem solving. They discussed their problems and discovered useful strategies. Teachers guided and instructed students throughout the discussion on how to solve problems efficiently and effectively.

### Traditional group

Students in the control group were taught using a marker and whiteboard. They participated in the exercises given by the teachers. The exercises are based on reference books provided by the school. Each school uses different reference books. Teachers narrated and jotted down information on the whiteboard. The enhanced educational curriculum unit requires every teaching method to be contextual. Thus, all teachings conducted in low secondary schools are traditionally contextual teaching.

### Training for teachers

Six teachers were involved in the RME approach. They were selected based on the criteria of the RME approach training organised by the Ministry of Education in Indonesia. The teachers underwent training for one month to ensure the success of the study and consistency with the design plan. The study objectives, RME and traditional approaches, planning and execution process and assessment methods were introduced to the teachers. The same teachers were assigned to treatment and control groups. The study was conducted after they understood the entire concept. The researcher observed throughout the study to determine whether the teachers were using the RME approach. Observation began from the start until the end of class for every session. The teachers were given feedback about their teaching. The researcher observed the traditional class to ensure that the teachers were not using the RME approach or any other teaching method.

### Pilot study

The present study was reviewed and approved by the Ministry of Education Pekanbaru City, Riau, Indonesia. A pilot study was conducted with 100 students to determine the validity and reliability of the research instrument. The validity of the research instrument was verified by four experts; two experts for content and two for language. According to the experts, the instrument language is suitable for measuring mathematical belief, representation and problem solving. The data from the pilot study were analysed using SPSS 23.0 and ANATES 4. Findings showed that the reliability of the mathematical belief instrument, problem solving and mathematical representation are 0.93, 0.87 and 0.80, respectively. The discriminant and difficulty index for the mathematical belief test and the mathematical problem solving test are at good and an average levels, respectively. [36] stated that the difficulty index value is at its best when used at the average level. The discriminant index should be at good and very good levels. The pilot study results indicated that the developed items are solid and strong for the actual study.

### Measure

#### Mathematical belief instrument

The instrument of mathematical belief was adapted from the Mathematical Problem Solving Beliefs Instrument [41] and students’ mathematics-related beliefs questionnaire [42]. The latter measures three factors of students’ mathematical belief, which are related to students in terms of mathematics students, mathematical discipline, mathematical teaching and learning. Sixty statements in the mathematical belief scale were used. Each statement could be answered with five responses of strongly agree (SA), agree (A), slightly disagree (SD), disagree (D) and strongly disagree (SLD).

#### Mathematical representation instrument

The instruments for mathematical representation consisted of a written test set with four questions on the topic of arithmetic. The instrument was constructed by the researcher to collect information about a representation problem solved by the students and their success in solving mathematical problems. This instrument had four problem statements with an open-question format. These mathematical problems required students to apply comprehension, analysis and interpretation in the context of daily life. The full score for each item was 4 and 0 was the lowest score.

#### Problem solving instrument

The Mathematical Problem Solving Beliefs Instrument is used to collect information about the method and the success of how the students solve mathematical problems. This instrument has five problem statements with an open-question format and requires students to comprehend, analyse and interpret these problems in the context of daily life. The full score for each item is 4 and 0 is the lowest score. The problem solving instrument is measured using marking schemes. The full score for each item is 4 and 0 is the lowest score. The total score of the students is changed to a scale of 0 to 100. The marking scheme for each item is shown in Table 3.

**Table 3:** Marking scheme for representation test and mathematical problem solving.

Score	Solution level
0	No effort, unclear answer, zero or not enough to be given a score
1	Some effort but wrong response
2	Mention problem setting using symbols in mathematical statement, graphics/table, algebra notes inefficiently, correctly and precisely and manage to obtain some correct or incomplete solutions
3	Mention problem setting using symbols in mathematical statement, graphics/table, algebra notes effectively, correctly and precisely and manage to obtain correct solutions
4	Mention problem setting using symbols in mathematical statement, graphics/table, algebra notes very effectively, correctly and precisely and manage to obtain correct solutions

The marking scheme used for levels of mathematical representation and problem solving is the same as that used by [43], which was adapted to the arrangement outlined by the government.

### Data analysis

The analysis for the actual study was performed using SPSS 23.0 and Amos 18. Analysis of covariance (ANCOVA) was performed to identify the difference in mathematical belief, representation and problem solving between the treatment and the control groups where the pre-test is a covariate. This step was followed in the structural equation modelling (SEM) test to identify the role of mathematical representation as a significant mediator in the relationship between mathematical belief and problem solving.

## Research findings

### Difference in mathematical belief gain score between treatment and control groups

Univariate Analysis of Variance (UNIANOVA) was performed to identify the gain scores of the mathematical belief of the treatment and the control groups. Certain requirements for the test needed to be met prior to UNIANOVA. These requirements include normality and homogeneity of variance between groups. The normality test showed the skewness and kurtosis values for the mathematical belief gain score for the treatment and the control groups are (0.07, -0.82) and (-0.36, 0.32), respectively. This result shows that normality requirement was met and data were considered normal if the skewness and kurtosis value ranged from -1.96 to +1.96 [44]. Therefore, one-way UNIANOVA can be performed to identify the differences in the mathematical belief gain score of the treatment and the control groups, as shown in Table 4.

**Table 4:** UNIANOVA: Difference in mathematical belief gain score of treatment and control groups.

Source	Type III Sum of Squares	Df	Mean Square	F	Sig.	Partial Eta Squared
Corrected Model	43.712^a^	1	43.712	39.963	0.000	0.084
Intercept	36.624	1	36.624	33.483	0.000	0.072
Group	43.712	1	43.712	39.963	0.000	0.084
Error	474.716	434	1.094			
Total	555.421	436				
Corrected Total	518.428	435				

a. R Squared = 0.084 (Adjusted R Squared = 0.082)

The UNIANOVA test result in Table 4 shows a significant difference in the mathematical belief gain score between the treatment and the control groups [F = 39.963, sig = 0.000 (p < 0.05)]. Students in the treatment group (mean = 0.606, std. error = 0.07) have a higher mathematical belief than students in the control group (mean = -0.027, std. error = 0.07). This finding means that the RME approach has better effect on the increase in the mathematical belief of students than the use of the traditional method. This differential effect size is medium (Cohen’s d = 0.61) [45]. Inspection of the 95% confidence intervals around each mean indicated that a significant increase in mathematical belief for participants in the treatment group and no increase in mathematical belief for participants in the control group, as shown in Table 5.

**Table 5:** Means, standard errors and 95% confidence interval in mathematical belief gain scores of treatment and control groups.

Group	Mean	Std. Error	95% Confidence Interval	
			Lower Bound	Upper Bound
Treatment	0.606	0.071	0.468	0.745
Control	-0.027	0.071	0-.166	0.113

**Fig 2:**
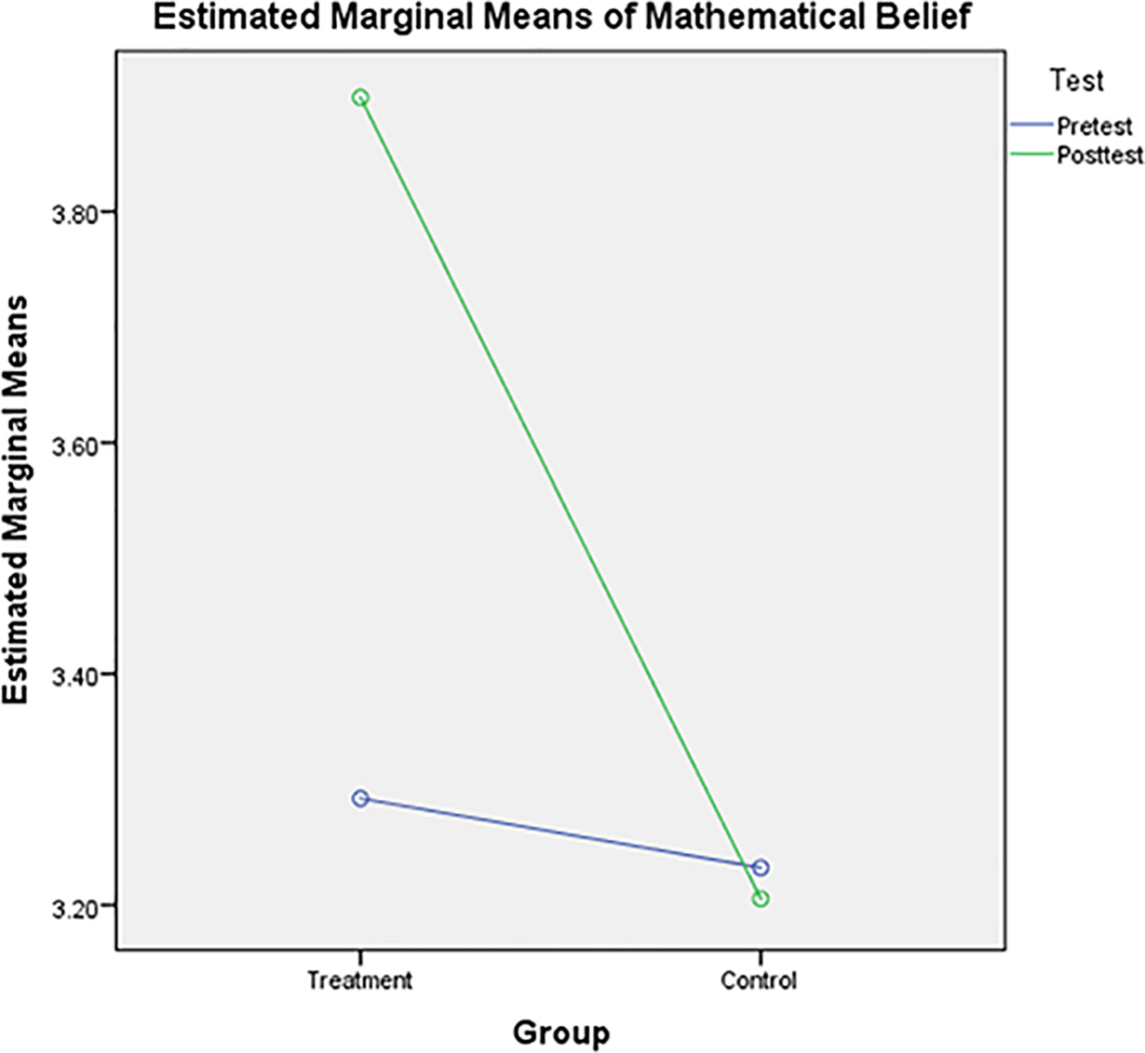
Mathematical belief means for the treatment and control groups.

Fig 2 shows the pre- and post-test means for a two-group design. In the treatment group, post-test results (mean = 3.90) had higher mathematical belief than pre-test results (mean = 3.29). However, in the control group, pre-test results (mean = 3.23) had higher mathematical belief than post-test results (mean = 3.21).

### Difference in mathematical representation gain score of treatment and control groups

UNIANCOVA was performed to identify the difference between the mathematical representation gain score of the treatment and the control groups. The normality test showed the skewness and kurtosis values for mathematical representation pre-test for the treatment (0.09, -0.57) and the control (-0.05, -0.78) groups. These results indicated that the normality requirement was met. Levene’s test obtained F = 1.525, sig = 0.434 (p > 0.05), which showed that the data had similar variances between groups. Thus, UNIANCOVA can be performed to identify the difference in mathematical representation gain scores between the treatment and the control groups.

The UNIANCOVA test result in Table 6 showed no significant difference between the mathematical representation gain score of the treatment and the control groups [F = 0.430, sig = 0.512 (p > 0.05)]. The mathematical representation gain score of the students in the treatment group (mean = 1.17) was similar to that of the students in the control group (mean = 1.23). This result indicated that the RME approach and the traditional method had the same effect on the increase in the mathematical representation of students. This differential effect size was small (Cohen’s d = 0.06) [45].

**Table 6:** UNIANCOVA: Differences in mathematical representation gain scores of treatment and control groups.

Source	Type III Sum of Squares	Df	Mean Square	F	Sig.	Partial Eta Squared
Corrected Model	0.294^a^	1	0.294	0.430	0.512	0.001
Intercept	626.329	1	626.329	916.310	0.000	0.679
Group	0.294	1	0.294	0.430	0.512	0.001
Error	296.654	434	0.684			
Total	923.166	436				
Corrected Total	296.948	435				

a. R Squared = 0.001 (Adjusted R Squared = -0.001)

**Fig 3:**
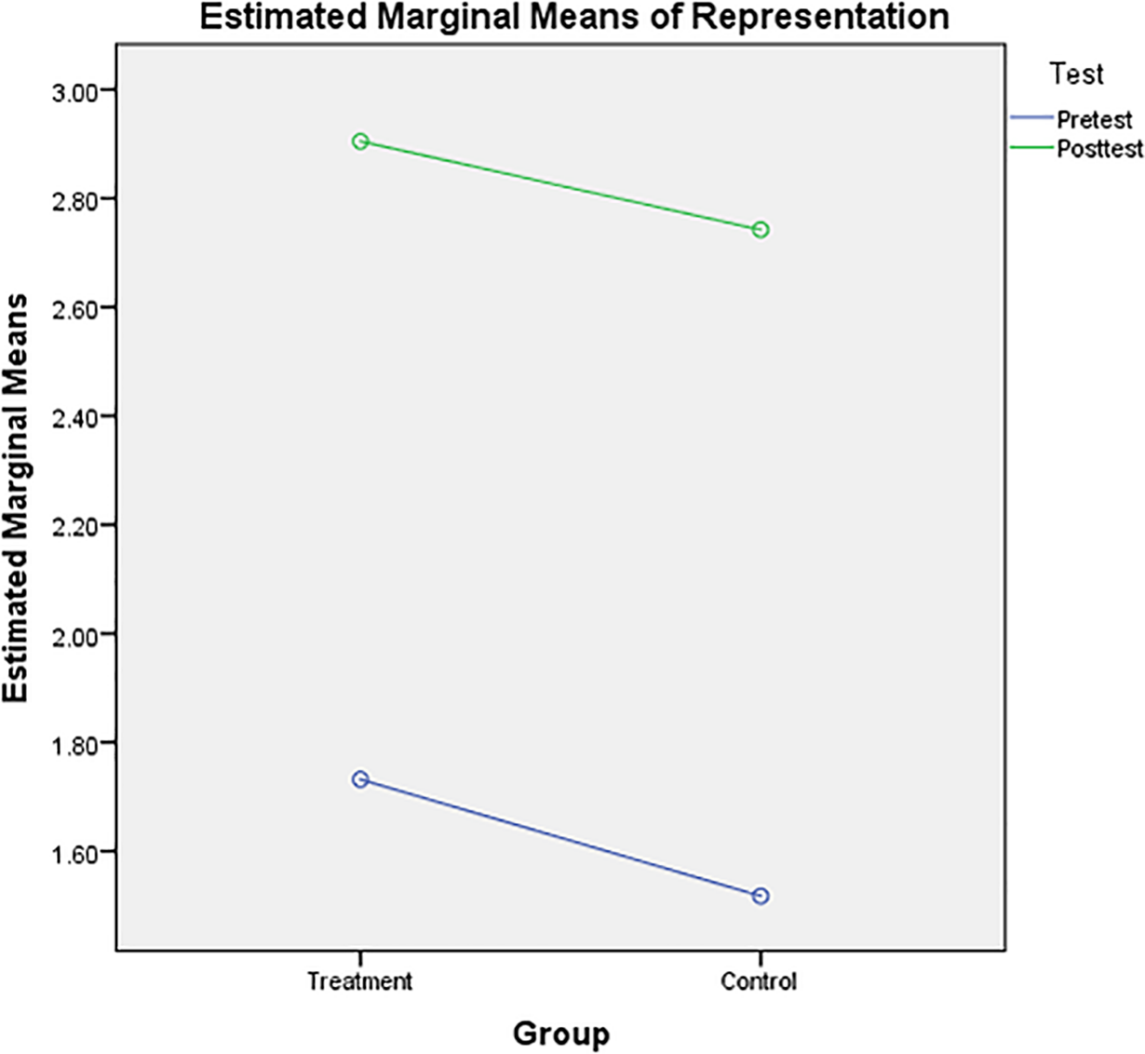
Mathematical representation means for the treatment and control groups.

Fig 3 shows the pre- and post-test means for a two-group design. In the treatment group, post-test results (mean = 2.90) had higher mathematical representation than pre-test results (mean = 1.73). However, in the control group, post-test results (mean = 2.74) had higher mathematical representation than pre-test results (mean = 1.52).

### Differences in mathematical problem-solving gain scores of treatment and control groups

UNIANCOVA was performed to identify the difference between mathematical problem-solving gain scores of the treatment and the control groups. The normality test showed the skewness and kurtosis values of mathematical problem-solving gain scores for the treatment group (-0.27. -0.81) and the control group (0.38, -0.48). Results showed that the normality requirement was met. Levene’s test obtained a value of F = 1.440, sig = 0.231 (p > 0.05), which indicated that the data had similar variances between groups. Therefore, UNIANCOVA can be performed to identify the differences between mathematical problem-solving gain scores of the treatment and the control groups, as shown in Table 7.

**Table 7:** UNIANCOVA: Differences in mathematical problem solving gain scores between treatment and control groups.

Source	Type III Sum of Squares	Df	Mean Square	F	Sig.	Partial Eta Squared
Corrected Model	3.010^a^	1	3.010	6.716	0.010	0.015
Intercept	1620.467	1	1620.467	3615.320	0.000	0.893
Group	3.010	1	3.010	6.716	0.010	0.015
Error	194.528	434	0.448			
Total	1818.681	436				
Corrected Total	197.539	435				

a. R Squared = 0.015 (Adjusted R Squared = 0.013)

The UNIANCOVA test result in Table 7 showed a significant difference in mathematical problem-solving gain scores between the treatment and the control groups [F = 6.716, sig = 0.010 (p < 0.05)]. Students in the treatment group (mean = 2.01) had better mathematical problem solving gain scores than the students in the control group (mean = 1.85). These results prove that the RME approach was better than the traditional method at improving problem solving skills. Such differential effect size was small (Cohen’s d = 0.25) [45].

**Fig 4:**
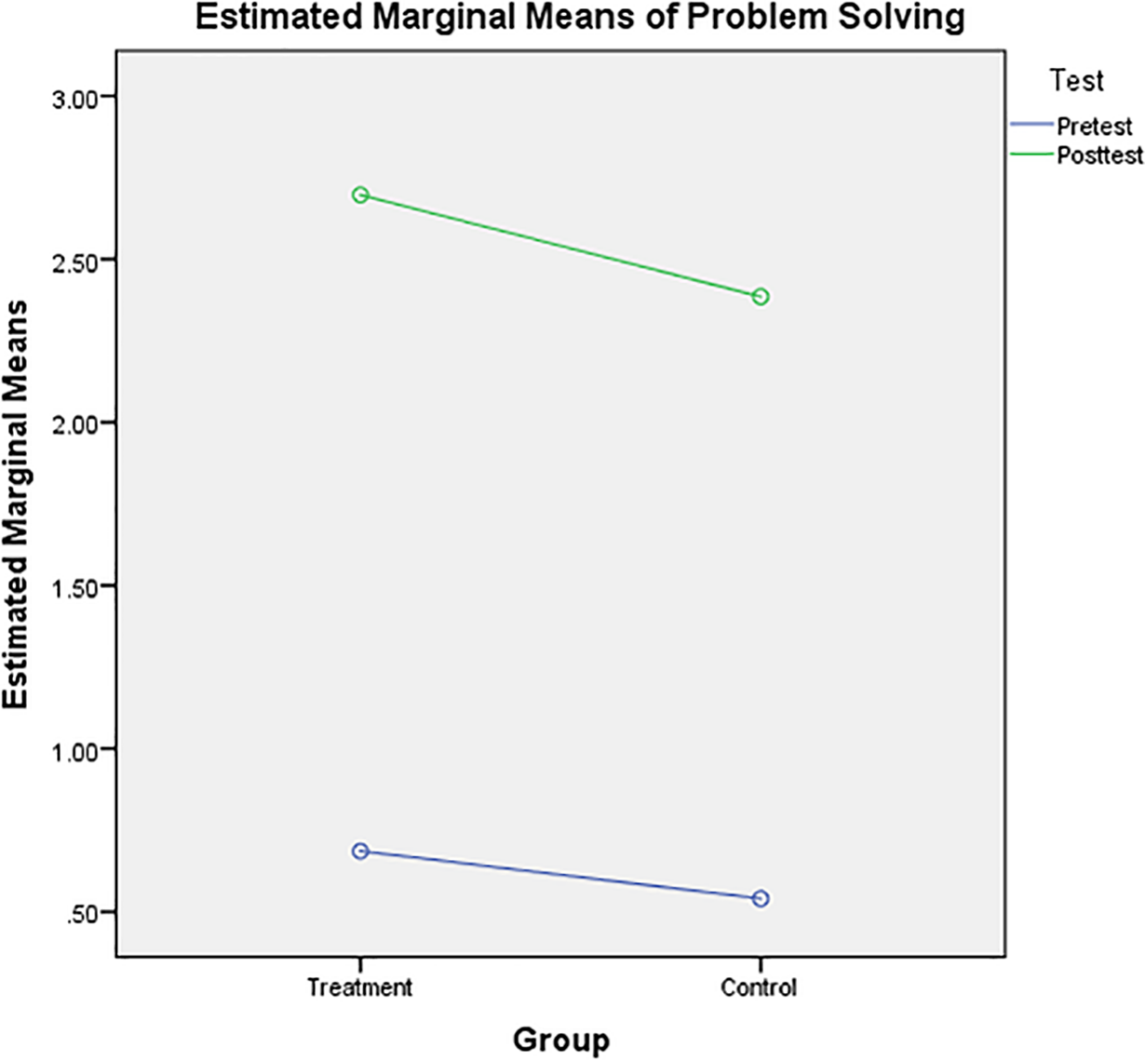
Mathematical problem-solving means for the treatment and control groups.

Fig 4 shows the pre- and post-test means for a two-group design. In the treatment group, post-test results (mean = 2.70) had higher mathematical problem-solving value than pre-test results (mean = 0.68). However, in the control group, post-test results (mean = 2.39) had higher mathematical problem-solving values than pre-test results (mean = 0.54).

### Role of mathematical representation as a mediator between mathematical belief and problem solving for the treatment group

**Fig 5:**
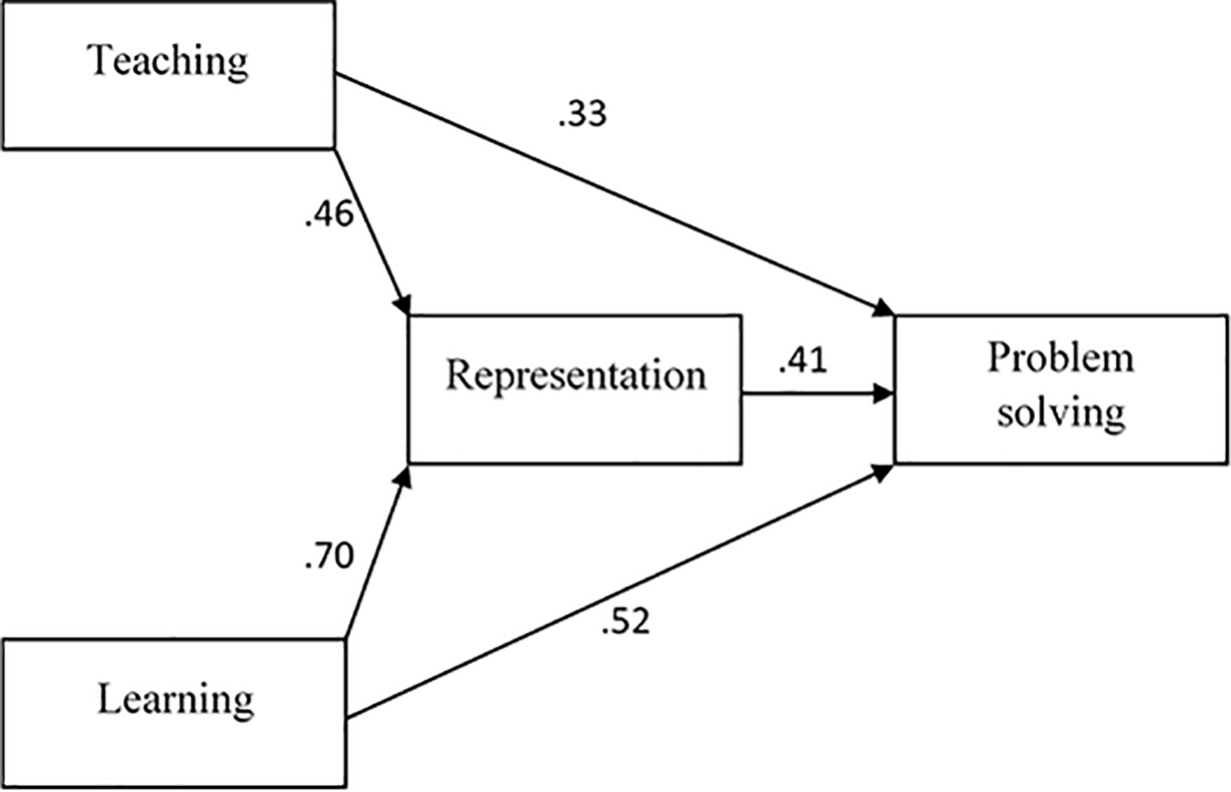
Role of mathematical representation as a mediator between mathematical belief and problem solving for the treatment group.

SEM analysis was performed to identify the role of arithmetic representation as a mediator between belief towards mathematical teaching and learning and mathematical problem solving. The analysis result of the SEM path model in Fig 5 shows the following: chi square/df = 3.06, root mean-square error approximation (RMSEA) = 0.07, goodness of fit index (GFI) = 0.91, Tucker–Lewis fit index (TLI) = 0.90 and comparative fit index (CFI) = 0.92. All assessments indicated that the data in the study had reasonable adjustment for the suggested model [46]. The result of SEM analysis showed that the suggested regression model was suitable when mathematical teaching belief (β = 0.33, p < 0.05) and mathematical learning belief (β = 0.52, p < 0.05) are significant predictor variables for mathematical problem solving. The SEM result showed that mathematical teaching belief (β = 0.52, p < 0.05) and mathematical learning belief (β = 0.70, p < 0.05) are significant predictor variables for arithmetic representation. Bootstrapping test was performed to determine the effect of mathematical representation as a significant mediator.

Bootstrapping test was applied to determine the effect of arithmetic representation as a significant mediator between mathematical teaching and learning belief and problem solving. Table 8 shows that arithmetic representation is a significant partial mediator between teaching belief (β = 0.19, p < 0.05) and learning (β = 0.29, p < 0.001) towards problem solving.

**Table 8:** Bootstrapping: Role of mathematical representation as a mediator between mathematical belief and problem solving in the treatment group.

Mediator	Direct effect	Indirect effect	Result
TB → MR → PS	0.33***	0.19*	Partial mediation
LB → MR → PS	0.51***	0.29***	Partial mediation

*** = significant at 0.001 level

* = significant at 0.05 level

### Role of mathematical representation as a mediator between mathematical belief and problem solving for the control group

**Fig 6:**
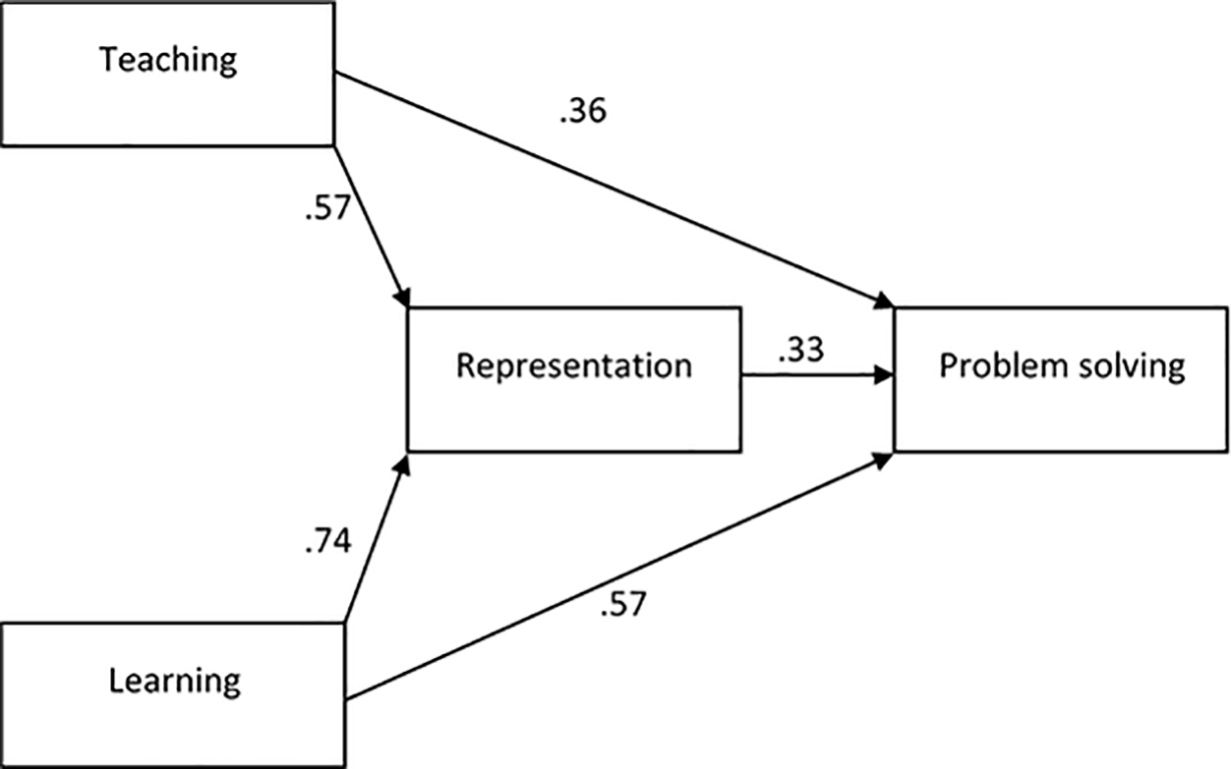
Role of mathematical representation as a mediator between mathematical belief and problem solving for the control group.

SEM analysis was performed to identify the role of arithmetic representation as a mediator between the belief towards mathematical teaching and learning in mathematical problem solving. The analysis of the SEM path model in Fig 6 shows the measure of chi square/df = 1.31, RMSEA = 0.07, GFI = 0.91, TLI = 0.90 and CFI = 0.92. The result of SEM analysis indicated that the suggested regression model was suitable when mathematical teaching belief (β = 0.36, p < 0.05) and mathematical learning belief (β = 0.57, p < 0.05) were significant predictor variables for mathematical problem solving. The SEM result showed that mathematical teaching belief (β = 0.57, p < 0.05) and mathematical learning belief (β = 0.74, p < 0.05) were significant predictor variables for arithmetic representation. Bootstrapping test was conducted to determine the effects of mathematical representation as a significant mediator (Table 9).

**Table 9:** Bootstrapping test: Role of mathematical representation as a mediator between mathematical belief and problem solving in the control group.

Mediator	Direct effect	Indirect effect	Result
TB → MR → PS	0.36***	0.19***	Partial mediation
LB → MR → PS	0.57***	0.25***	Partial mediation

*** = significant at .001 level

The bootstrapping test was applied to check the effect of arithmetic representation as a significant mediator between mathematical teaching and learning belief and problem solving. Table 9 shows that arithmetic representation was a significant mediator for teaching belief (β = 0.19, p < 0.001) and learning (β = 0.25, p < 0.001) towards problem solving. The SEM result indicated that the treatment and the control groups obtained the same results for the role of mathematical representation as a partial mediator between mathematical belief and problem solving.

## Discussion

Students who were taught using the RME approach had higher mathematical belief than students who were exposed to the traditional method. The use of RME increased the confidence of students in mathematics, especially in arithmetic, as reflected in their active participation in the activities presented with the RME approach. According to Fauzan [47], active students use the RME approach, which develops creative thinking and lessens uncertainty towards mathematics. However, the use of the traditional method successfully increased the mathematical belief of students, although the RME approach had better effect. Saragih [48] stated that the advantage of the RME approach is its ability to strengthen students’ interest in mathematics. The findings supported Lee, Zeleke and Mavrotheris [30] who asserted that the RME approach enables students to learn mathematics actively such that their belief can increase through the effort of teachers. Greer, Verschaffel and de Corte [31] supported this idea by stating that the mathematical belief of students is influenced by factors, such as teachers, textbooks, learning strategies and use of problems that exist in the surroundings of students for learning activities.

The use of the RME approach did not significantly increase mathematical representation compared with the traditional method. Thus, the RME approach was not suitable for all skills or topics. However, the RME approach still successfully increased the mathematical representation of students. This idea was supported by Arsaythamby and Zubainur [49] who claimed that not all learning activities of students should be conducted using the RME approach. Teaching with the RME approach provided students with the opportunities to come up with ideas that can enable them to solve mathematical problems easily. The traditional method provided opportunities for students to generate ideas, but these opportunities are fewer than those offered by the RME approach. Neria and Amit [50] mentioned that questions on mathematical representation are given to students to allow them to present situational problems in the form of mathematical notes, numerals, symbols, graphics, tables and pictures, which they will try to solve later. Therefore, the skills of teachers in using the RME approach must increase the mathematical representation of students to guide their gradual learning according to levels.

The RME approach successfully improved the problem-solving skills of students and was better than the traditional method in this aspect. In the RME approach, teachers checked the answers of students by writing down detailed answers and providing reasons or explanations as to how the answer was obtained. Moreover, students were motivated to stand in front of the class and explain their work. Jones, Thornton and Nisbet [51] found that the RME approach is suitable for arithmetic learning until the students become more confident in solving problems. This statement supported the findings of Viholainen, Asikainen and Hirvonen [52], who stated that confidence in mathematics has strong influence on mathematical problem solving and determines how a student chooses the approach, technique and strategy to use. The results of study supported Laurens, Batlolona, Batlolona and Leasa [4], who claimed students who were taught with RME achieved better results than the students who were involved in conventional learning.

The SEM test showed the same match between the treatment and the control groups, wherein mathematical representation was a significant partial mediator between mathematical belief and problem solving. Findings showed that mathematical belief indirectly affected mathematical problem-solving skills. This study indicated no significant difference in mathematical representation, but the mediator effect of mathematical representation between treatment and control groups was the same. This result suggests that mathematical representation is an indirectly important aspect in students to enhance the relationship between mathematical beliefs and problem solving. The use of different methods did not influence the effect of mathematical representation as the mediator of the relationship between mathematical beliefs and problem solving. The findings supported Hwang, Chen, Dung and Yang [15] in their claim that mathematical representation contributes to the ability of students to solve mathematical problems. This study supported Ling and Ghazali [26], who reported that arithmetic is the most frequently used representation model by students in problem solving, including answer verification from all the given questions. Moreover, mathematical belief affects the mathematical representation and problem-solving skills of students. This finding means that if students believe in mathematical teaching and learning, then they will possess mathematical representation and reliable problem-solving skills. This statement is consistent with the findings of [3], who found that the belief of students towards mathematics can influence their view on mathematical discipline, which is related to mathematical teaching and learning. The SEM results showed higher connection of mathematical belief and mathematical representation in problem solving with the use of the RME approach than with the use of the traditional method. This finding is supported in Muchlis [53] and in Husna and Saragih [54].

The study successfully proved that the RME approach had a positive effect on mathematical belief, representation and problem solving among students. Thus, teachers need to adjust their teaching methods using RME and encourage students to participate in activities and engage in discussions. The RME approach provides students with the opportunity to generate knowledge on the topics that they have been taught. Students can convey their ideas until they can form concepts for each learning step. Many students provide solutions that consist of different steps but have the same answer. Students believe in producing results that they obtain by themselves, which is a process that they will later find as an arithmetic concept. School administrators must assist teachers in eliminating the negative perception towards teaching and learning mathematics. The effectiveness of RME offers an opportunity to use the approach continuously to teach other topics for secondary school students as a whole. Future studies can examine the use of RME at various educational levels to obtain detailed information.

The contribution of this study is the identification of various learning methods often used by students in everyday life that can be utilised to improve the quality in learning through the creativity of teachers. In additional, the RME approach is among the most effective approaches in fostering mathematical representation, belief and problem-solving skills that could improve student achievement. Few studies examined the relationship of mathematical representation as a mediator between mathematical belief and problem solving. The present study filled the gap by producing a new form of relationship model through a quasi-experimental design.

The findings and results of this study provided information on the differences in mathematical representation, belief and problem-solving skills of students who learned through RME and conventional learning methods. Mathematics teachers should apply RME in the classroom to make abstract mathematical concepts more understandable. Teachers should be creative and innovative in designing learning with this approach. Teachers should develop additional learning media, strategies or models that are more suitable with learning materials or with the contexts of students. Further, schools should create contextual environments that are rich in information on ways to solve real life problems.

## Conclusion

The use of RME can increase mathematical belief, representation and problem solving skills. This approach successfully trains students to formulate their own ideas from real-life situations or experiences. Teachers must be encouraged to use the RME approach in teaching and learning mathematics. Efforts pertaining to mathematical representation should be doubled to increase the mathematical problem solving skills of students. The belief of students is another major factor in increasing mathematical problem solving skills. Cooperation from all sides should be improved to encourage the use of the RME approach in teaching and learning mathematics at all school levels to increase mathematical belief, representation and problem solving. This study seeks to serve as a stepping stone for future studies to expand the use of the RME approach from the national to the international level.

## Supporting information

S1 File Data availability. (XLS)
